# Clinical and Radiological Outcomes of Full-Endoscopic Decompression for Lumbar Spinal Stenosis With Grade I Degenerative Spondylolisthesis: A Retrospective Study With a Minimum 1-Year Follow-up

**DOI:** 10.1227/neuprac.0000000000000078

**Published:** 2024-01-11

**Authors:** Sang-Soo Choi, Geon Ahn, Il-Tae Jang, Hyeun Sung Kim

**Affiliations:** ‡ Department of Spine Surgery, Nanoori Gangnam Hospital, Seoul, South Korea;; § Department of Spine Surgery, Harrison Spinartus Hospital Chungdam, Seoul, South Korea

**Keywords:** Spondylolisthesis, Full-endoscopic decompression

## Abstract

**BACKGROUND AND OBJECTIVES:**

Decompression without fusion is a surgical option for the treatment of lumbar spinal stenosis (LSS) with grade I degenerative spondylolisthesis (DS). However, the outcomes of full-endoscopic decompression remain unknown. This study aimed to investigate the clinical and radiological outcomes of lumbar full-endoscopic decompression for spinal stenosis with grade I DS.

**METHODS:**

A total 43 patients with LSS with grade I DS who underwent lumbar full-endoscopic unilateral laminotomy for bilateral decompression and were followed up for a minimum of 1 year were retrospectively reviewed. Data on demographic characteristics, operative details, radiological images, clinical outcomes, and complications were analyzed.

**RESULTS:**

Pain, evaluated using the visual analog scale, was significantly reduced in patients undergoing full-endoscopic decompression in both the leg and back; the Oswestry Disability Index scores also improved (*P* < .001). MacNab's outcome classification was excellent or good in 95% of patients. One patient eventually needed the fusion surgery, and two patients required further diskectomy surgery because of recurrent symptoms. During follow-up, an increase in the vertebral slip was observed in 46.5% of the patients, but it was not statistically significant (*P* = .46). The progression of DS, defined as percentage of slip >5%, was observed only in 7% of the patients. An increase in vertebral slip was not associated with the clinical outcomes. There was no difference in radiological outcomes, such as angular motion (*P* = .36) and translation (*P* = .13), compared with the preoperative state. The operation did not exacerbate spinal instability. Rather, fewer patients had spinal instability postoperatively.

**CONCLUSION:**

Endoscopic decompression alone has comparably good clinical outcomes with those of other surgical techniques in patients with LSS and DS. Few postoperative spinal instability occurred. Rather, the number of patients with instability decreased postoperatively. Full-endoscopic decompression may be a treatment option for patients with grade 1 DS.

**ABBREVIATIONS:**
DS: degenerative spondylolisthesis
LE-ULBD: lumbar full-endoscopic unilateral laminotomy for bilateral decompression
LSS: lumbar spinal stenosis
MCIDs: minimal clinically important differences
ODI: Oswestry Disability Index.

Lumbar spinal stenosis (LSS) is the most common lumbar degenerative disease in the elderly population^1^ and often occurs simultaneously with degenerative spondylolisthesis (DS), which has a significant effect on a patient's quality of life. Traditionally, DS is treated using decompression with fusion.^2,3^ In the Spine Patient Outcomes Research Trial, better outcomes were noted for fusion at a follow-up of 4 years.^4,5^ Furthermore, some studies have suggested that reoperation rates are higher for laminectomy alone than for laminectomy plus fusion.^4-6^ However, recent studies have challenged this belief. Butler et al^5^ and Carlsson et al^7^ reported similar clinical outcomes when comparing decompression alone with decompression with fusion in patients with low-grade DS. Kuo et al^8^ reported that the reoperation rates of unilateral laminotomy for bilateral decompression are comparable with those of posterior decompression and fusion. However, the most appropriate surgical option for the treatment of low-grade DS remains unclear.

In recent years, minimally invasive spinal surgery has become popular because it does not require extensive dissection of the multifidus muscle and resection of the spinous process and interspinous ligaments, which may cause spinal instability and postoperative back pain.^9^ Full-endoscopic spine surgery is one of the most minimally invasive procedures to date, with better features, such as tiny skin incisions, minor soft tissue injuries, less postoperative pain, shorter hospitalization, and earlier return to social life, than conventional open surgery.^10^ In particular, because full-endoscopic spinal surgery can preserve tensegrity almost perfectly, it can be inferred that the progress of instability can be minimized and it may even help recovery from instability.

This state-of-the-art surgical procedure does not have as much clinical data as conventional open surgery, especially for LSS with grade I DS. Several authors have reported good clinical outcomes with full-endoscopic decompression for LSS.^11,12^ Others have reported good results of full-endoscopic decompression in patients with LSS with or without DS who maintained spinal stability.^13,14^ Nevertheless, there are still no clinical or radiological outcome data for patients with DS, with or without spinal instability.

In this context, we retrospectively evaluated the clinical and radiological outcomes of patients with LSS and grade I DS, with or without spinal instability. We hypothesized that full-endoscopic decompression has comparable clinical results and radiological outcomes with conventional treatment and that it preserved tensegrity and does not cause iatrogenic instability or DS progression. Interestingly, our hypothesis went further than what we expected. To the best of our knowledge, this is the first study to report the radiological results of full-endoscopic lumbar decompression.

## METHODS

### Study Design and Patient Sample

We retrospectively reviewed 57 patients with Grade I DS who underwent lumbar full-endoscopic unilateral laminotomy for bilateral decompression (LE-ULBD) in a single institution between February 2018 and December 2019. The surgical criteria were as follows: symptomatic LSS with Grade I DS manifesting as lumbar radiculopathy with or without back pain or neurogenic claudication and failed conservative treatment for at least 3 months. The exclusion criteria were as follows: revision surgery at the same level, back pain without radiculopathy, DS > grade I, infection, LSS combined with fractures, or loss to follow-up (less than 1-year follow-up). Fourteen patients were excluded based on these criteria, and 43 were included in the analysis.

### Definitions of Outcome Variables

The patients were retrospectively evaluated using medical records, standing plain radiography, and MRI. The clinical outcomes were measured before surgery, after surgery, 3 months after surgery, and at the final follow-up using the visual analog scale (VAS) for low back pain and lower limb pain and Oswestry Disability Index (ODI) for assessing functions in activities of daily living. The minimal clinically important differences (MCIDs) of the VAS and ODI were defined as 3 and 12 points, respectively. MacNab's criteria were used for assessment at the final follow-up.

LSS was determined using MRI, and DS was assessed by measuring the percentage of slip from plain standing radiographs. The percentage of slip was defined as follows:

$$\text{Percent}\;\text{slip}=\frac{\text{distance}\;\text{between}\;\text{post}\;\text{edge}\;\text{of}\;\text{adjacent}\;\text{vertebral}\;\text{bodies}}{\text{length}\;\text{of}\;\text{sup}\;\text{endplate}\;\text{of}\;\text{inf}\;\text{vertebral}\;\text{body}}$$

Translation and angular motion were also measured to determine dynamic spinal stability. Translation was defined as the difference in slip between flexion and extension on plain dynamic radiographs. Angular motion is the difference in the angle of the superior endplate of the adjacent vertebral bodies between flexion and extension. Radiological spinal instability was defined as translation of >4 mm or angular motion of >10°.^15-17^ These radiological data were measured by two neurosurgeons separately, and the average values were calculated.

The radiological progression of spondylolisthesis was defined as a percentage of slip increase of more than 5%.^9,14,17^ The radiological progression of instability was defined as an increase in translation or angular motion that eventually met the criteria or exceeded the previously met criteria.

### Surgical Technique of LE-ULBD

**FIGURE 1.**
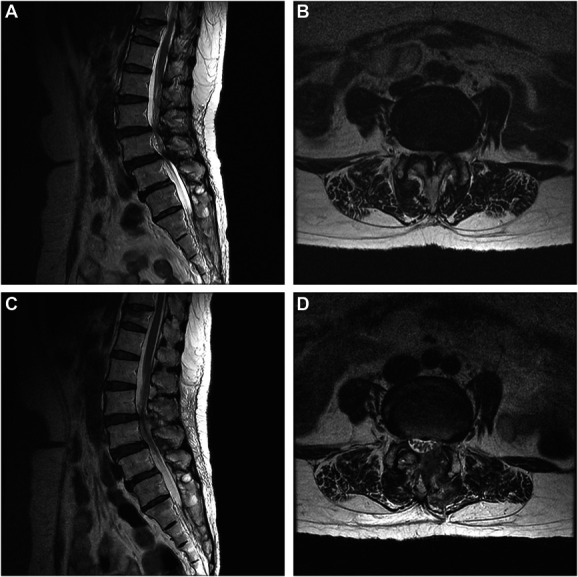
MR images of a 58-year-old female who underwent full-endoscopic decompression at L3/4 and L4/5 levels for spinal stenosis with grade 1 spondylolisthesis. **A** and **B**, Preoperative images showing both central and lateral recess stenosis at the L4/5 level with grade I spondylolisthesis. **C** and **D**, Postoperative images in which decompression of the dural sac can be seen.

The patient was placed in prone position on a Wilson frame. Most patients were placed under conscious sedation and epidural anesthesia. Some patients received general anesthesia when it was inevitable. An iLESSYS Delta Endoscopic system (Joimax GmbH) was used for the surgery. It has a working cannula of 13.7 mm outer diameter and 10.2 mm inner diameter. The endoscope has a 10.0-mm, a 6.0-mm working cannula, and a 15-degree view angle. A Primado II high-speed drill system (NSK-Nakanishi International) was used to remove the bony structures. A bipolar radiofrequency electrocoagulator (Elliquence) was used for hemostasis and coagulation. A 1.5-cm incision was made on the lateral border of the interlaminar space at the level of the planned decompression. After docking the guidewire to the ipsilateral caudal lamina, consecutive dilations and introduction of the working channel were performed. After drilling the bony structures around the ipsilateral ligament flavum, its outer layer was removed. The contralateral boundary of the spinal canal was then exposed by drilling it in a sublaminar fashion. After exposure, we removed the outer layer of the contralateral ligamentum flavum, followed by removal of the ipsilateral and contralateral inner ligament flavum^18^ (Figure 1).

### Statistical Analysis

Descriptive data are expressed as means ± SD. Statistical analyses were performed using IBM SPSS Statistics for Windows (IBM Corp.) with paired and independent *t*-tests. Statistical significance was set at *P* < .05.

### Declarations

All data supporting the findings presented in this article are available upon reasonable request from the corresponding author. The study was conducted in accordance with the Declaration of Helsinki and approved by the Institutional Review Board. The informed consent is not required for this study because this research involves not more than minimal risk of harm to patients.

## RESULTS

### Baseline Characteristics

A total of 43 patients who met the eligibility criteria were included in this study. There were 13 (30.2%) male patients, and the median age was 64.4 (SD, 9.2) years. A total of 76.7% of patients received epidural anesthesia. The term “Level of decompression” indicates the level with grade I DS at which the patient underwent LE-ULBD. Thirty of the 43 cases occurred at the L4/5 level. The baseline characteristics of the patients are presented in Table 1.

**TABLE 1.** Demographic Data

Characteristics	Patients group (n = 43)
Male, n (%)	13 (30.2%)
Median age, y (SD)	64.4 (9.2)
Levels of decompression (No. of cases)	
L2/3	1
L3/4	10
L4/5	30
L5/S1	2
Single-level decompression (No. of cases)	19 (44.2%)
Operation time (min), mean (SD)	92.9 (36.8)
Duration from the onset of symptoms to surgery (mo), mean (range)	19.6 (3-120)
Type of anesthesia (No. of cases)	
General anesthesia	10 (23.3%)
Epidural anesthesia	33 (76.7%)
Duration of follow-up (mo), mean (range)	22.4 (12-54)
Preoperative comorbidities (No. of cases)	
Hypertension	14
Diabetes mellitus	9
Cardiovascular diseases	11
Chronic kidney diseases	2
Respiratory diseases	2
Other diseases	4

### Clinical Outcomes

The mean VAS score for leg pain was significantly lower postoperatively (3.1 ± 0.4, *P* < .001), 3 months (2.4 ± 0.6, *P* < .001) after surgery, and at the final follow-up (2.1 ± 0.9, *P* < .001), than that preoperatively (7.7 ± 1.2). The average VAS score for back pain was significantly lower postoperatively (3.0 ± 0.5, *P* < .001), 3 months after surgery (2.3 ± 0.6, *P* < .001), and at the final follow-up (2.1 ± 0.8, *P* < .001) than that preoperatively (7.2 ± 1.1). The ODI scores improved significantly postoperatively (31.3 ± 3.9, *P* < .001), 3 months after surgery (26.4 ± 4.2, *P* < .001), and at the final follow-up (24.7 ± 5.4, *P* < .001) compared with the preoperative ODI scores (74.6 ± 7.2). At the final follow-up, there were MCIDs in VAS scores for leg and back pain in 95.3% and 97.7% of patients, respectively. MCIDs in ODI scores were found in 100% of patients. MacNab's criteria were applied and graded as excellent or good in 95.3% of the patients. Three cases of perioperative complications were identified: two patients had dural tears that were repaired in situ, but they developed symptoms of cerebrospinal fluid leakage; one patient developed postoperative epidural hematoma. The three cases of complications were successfully treated with conservative management (Table 2).

**TABLE 2.** Clinical Outcomes

Outcome	Patients group (n = 43)	*P* value	95% CI
VAS for leg pain			
Preoperative	7.7 ± 1.2		
Postoperative	3.1 ± 0.4	<.001	4.19–4.92
3 months postoperatively	2.4 ± 0.6	<.001	4.89–5.76
Final follow-up	2.1 ± 0.9	<.001	5.09–6.08
VAS for back pain			
Preoperative	7.2 ± 1.1		
Postoperative	3.0 ± 0.5	<.001	3.82–4.55
3 months postoperatively	2.3 ± 0.6	<.001	4.52–5.25
Final follow-up	2.1 ± 0.8	<.001	4.7–5.53
ODI			
Preoperative	74.6 ± 7.2		
Postoperative	31.3 ± 3.9	<.001	40.73–45.88
3 months postoperatively	26.4 ± 4.2	<.001	45.41–51.05
Final follow-up	24.7 ± 5.4	<.001	46.88–52.84
Macnab's criteria			
Excellent	12 (27.9%)		
Good	29 (67.4%)		
Fine	2 (4.7%)		
Poor	0 (0%)		
Complications			
Dura tear	2 (4.7%)		
Postoperative hematoma	1 (2.3%)		
Reoperation			
Fusion	1 (2.3%)		
Diskectomy	2 (4.7%)		

ODI, Oswestry Disability index; VAS, visual analog scale.

### Reoperation Cases

Two revision surgeries were performed during the follow-up period because of recurrent symptoms of restenosis because of disk herniation at the L4/5 level. One patient was treated with transforaminal endoscopic lumbar diskectomy, and the other was treated with interlaminar endoscopic diskectomy. Meanwhile, there was one case of revision fusion surgery at the L5/S1 level. The patient experienced recurrent back and radicular pain after surgery because of aggravation of spinal instability. Therefore, percutaneous full-endoscopic transforaminal lumbar interbody fusion at the L5/S1 level was performed, which relieved the patient's symptoms.

### Radiological Outcomes

**FIGURE 2.**
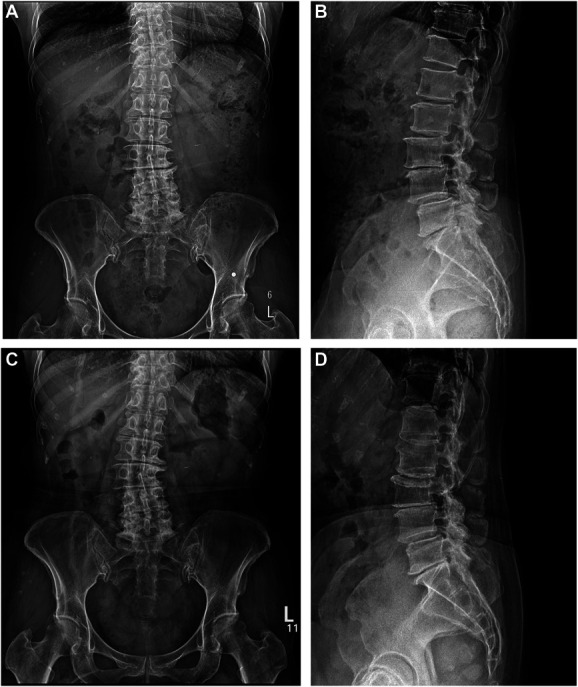
Plain x-ray images of a 66-year-old female who underwent full-endoscopic decompression at L3/4 and L4/5 levels for spinal stenosis with grade 1 spondylolisthesis. **A** and **B**, The preoperative images and **C** and **D**, the final follow-up images show decreased anterior slip of the L4 on L5 at the final follow-up.

Regarding the effects of endoscopic decompression on DS progression, there was no significant difference in percentage of slip between the baseline and final follow-up (10.75 ± 0.70 vs 11.17 ± 0.63, *P* = .46). Nearly half of the patients (46.5%) had increased percentage of slip at the final follow-up compared with the baseline. However, interestingly, the rest of the patients (53.5%) had a decreased percentage of slip (Figure 2). There were only three cases (7.0%) of radiological progression of DS, defined as an increase in percentage of slip >5%. However, there was no correlation between radiological progression of DS and clinical outcomes such as VAS scores, ODI scores, or MacNab's criteria outcome.

**FIGURE 3.**
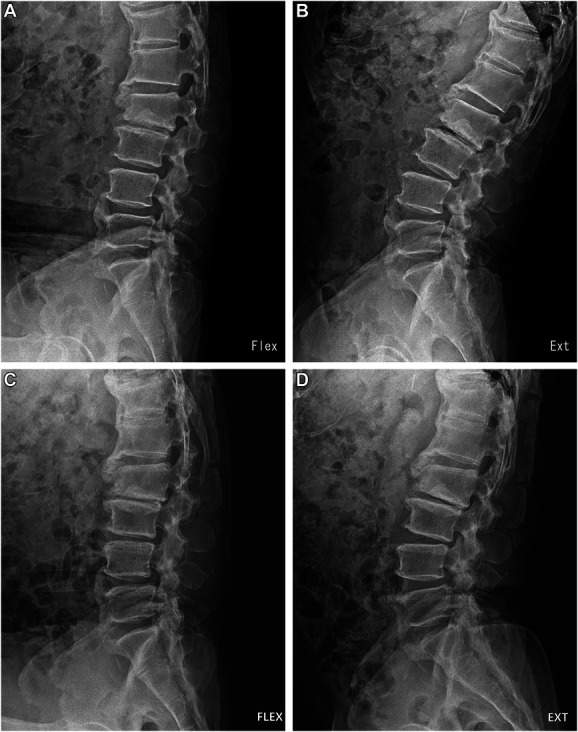
Dynamic x-ray images of a 64-year-old male who underwent full-endoscopic decompression at the L4/5 level for spinal stenosis with grade 1 spondylolisthesis. **A**, The preoperative flexion and **B**, extension images and **C**, the final follow-up flexion and **D**, extension images show a significantly improved angular instability at the final follow-up.

There were no significant differences in both the angular motion (7.91 ± 0.63 vs 7.40 ± 0.70, *P* = .356) and translation (2.20 ± 1.48 vs 1.83 ± 1.32, *P* = .127) between baseline and the final follow-up. There were 22 patients (51.2%) who had spinal instability at the baseline, but interestingly, this reduced to 12 patients (11.6%) at the final follow-up (Figure 3). Instability was aggravated in five (11.6%) patients, and one (2.3%) patient had new onset instability (Table 3).

**TABLE 3.** Radiological Outcomes

Outcome	Patient group (n = 43)
Percentage of slip (%)	
Preoperative	10.75 ± 0.70
Final follow-up	11.17 ± 0.63
*P*-value	0.460
95% CI	−1.53 to 0.7
Angular motion (flexion-extension) (°)	
Preoperative	7.91 ± 0.63
Final follow up	7.40 ± 0.70
*P*-value	0.356
95% CI	−0.6 to 1.63
Translation (flexion-extension) (mm)	
Preoperative	2.20 ± 1.48
Final follow up	1.83 ± 1.32
*P*-value	0.127
95% CI	−0.11 to 0.85
Increased percentage of slip (No. of cases)	20 (46.5%)
Decreased percentage of slip (No. of cases)	23 (53.5%)
Aggravation of spondylolisthesis (percentage of slip >5%) (No. of cases)	3 (7.0%)
Radiological instability	
Preoperative (No. of cases)	22 (51.2%)
Final follow up (No. of cases)	12 (27.9%)
Aggravation of instability (No. of cases)	5 (11.6%)
New onset of instability (No. of cases)	1 (2.3%)

### Subgroup Analyses

As we noticed from the radiological outcomes, 23 of 43 patients showed a decreased percentage of slip after the operation, which could indicate an improvement in DS. Therefore, we divided the patients into two groups to determine if there were any other differences between the two groups in addition to changes in the percentage of slip.

Table 4 shows the demographic data and clinical outcomes of the two groups. The increased percentage of the slip group (I group) had fewer male patients than the decreased percentage of the slip group (D group) (15% vs 43.5%, *P* = .044). Preoperative VAS scores for leg pain were slightly higher in the I group than in the D group (8.2 ± 1.1 vs 7.3 ± 1.1, *P* = .014). However, there were no differences in demographic characteristics and clinical outcomes such as VAS scores, ODI, and MacNab's criteria outcome.

**TABLE 4.** Clinical Outcomes in Subgroups

Outcome	Increased percentage of slip (n = 20)	Decreased percentage of slip (n = 23)	*P* value	95% CI
Male	3 (15.0%)	10 (43.5%)	.044	N/A
Median age (SD)	62.8 (10.9)	65.9 (6.8)	.273	−8.8 to 2.55
Levels of decompression				
L2/3	1	0	.887	N/A
L3/4	4	6	.648	N/A
L4/5	13	17	.887	N/A
L5/S1	2	0	.549	N/A
Single-level decompression	11 (55.0%)	11 (47.8%)	.242	N/A
Operation time (min), mean (SD)	88.6 (30.8)	95.0 (39.9)	.721	41.21 to 28.75
Duration from the onset of symptoms to surgery (mo), mean (range)	19.7 (3-120)	19.5 (3-60)	.981	−15.05 to 15.4
Type of anesthesia (No. of cases)				
General anesthesia	3 (15.0%)	7 (30.4%)	.242	N/A
Epidural anesthesia	17 (85.0%)	16 (69.6%)	.242	N/A
Duration of follow-up (mo), mean (range)	19.1 (12-48)	25.2 (12-54)	.169	14.71 to 2.66
VAS scores for leg pain				
Preoperative	8.2 ± 1.1	7.3 ± 1.1	.014	0.18–1.51
Postoperative	3.3 ± 0.4	3.0 ± 0.4	.103	−0.04 to 0.46
3 months postoperatively	2.4 ± 0.7	2.4 ± 0.6	.83	−0.43 to 0.35
Final follow-up	2.2 ± 0.9	2.1 ± 0.9	.823	−0.5 to 0.63
*P*-value	<0.001	<0.001		
95% CI	5.32–6.68	4.5–5.94		
VAS scores for back pain				
Preoperative	7.3 ± 0.9	7.2 ± 1.2	.821	−0.59 to 0.75
Postoperative	3.1 ± 0.6	3.0 ± 0.3	.365	−0.17 to 0.46
3 months postoperatively	2.4 ± 0.7	2.3 ± 0.5	.795	−3.1 to 0.39
Final follow-up	2.3 ± 0.8	2.0 ± 0.7	.204	−0.17 to 0.76
*P*-value	<0.001	<0.001		
95% CI	4.47–5.53	4.55–5.88		
ODI				
Preoperative	77.5 ± 5.6	72.1 ± 7.6	.012	1.24–9.59
Postoperative	32.1 ± 4.5	30.6 ± 3.3	.215	−0.9 to 3.88
3 months postoperatively	26.5 ± 4.9	26.3 ± 3.5	.853	−2.35 to 2.83
Final follow-up	24.8 ± 6.0	24.7 ± 4.8	.95	−3.24 to 3.45
*P*-value	<0.001	<0.001		
95% CI	48.8–56.6	42.96–51.83		
Macnab's criteria				
Excellent	5 (25.0%)	7 (30.4%)	.7	N/A
Good	14 (70.0%)	15 (65.2%)	.746	N/A
Fine	1 (5.0%)	1 (4.4%)	.922	N/A
Poor	0	0	N/A	
Complications				
Dura tear	1 (5.0%)	1 (4.4%)	N/A	
Postoperative hematoma	1 (5.0%)	0	N/A	
Reoperation				
Fusion	1 (5.0%)	0	N/A	
Diskectomy	1 (5.0%)	1 (5.0%)	N/A	

ODI, Oswestry Disability index; VAS, visual analog scale.

Table 5 presents the radiological outcomes of the two subgroups. As shown in Table 3, we noticed no overall difference in the percentage of slip before and after surgery. However, there were significant differences in the percentage of slip in each group (I group: 9.89 ± 3.98 vs 13.14 ± 5.72, *P* < .001) (D group: 11.50 ± 5.04 vs 9.45 ± 4.77, *P* < .001). By contrast, there were no differences in angular motion (7.06 ± 4.89 vs 7.69 ± 4.46, *P* = .66) or translation (1.85 ± 1.24 vs 1.81 ± 1.42, *P* = .932).

**TABLE 5.** Radiological Outcomes in Subgroups

Subgroups	Increased percentage of slip	Decreased percentage of slip	*P* value	95% CI
No. of patients	20	23		
Percentage of slip (%)				
Preoperative	9.89 ± 3.98	11.50 ± 5.04	.256	−4.44 to 1.22
Final follow-up	13.14 ± 5.72	9.45 ± 4.77	.026	0.46–6.92
*P*-value	<0.001	<0.001		
95% CI	−4.72 to −1.78	1.3–2.8		
△^a^	3.25 ± 3.13	−2.05 ± 1.74	<.001	3.77–6.84
Angular motion (flexion-extension) (°)				
Preoperative	7.74 ± 4.52	8.07 ± 3.81	.799	−2.89 to 2.24
Final follow up	7.06 ± 4.89	7.69 ± 4.46	.66	−3.51 to 2.25
*P*-value	0.404	0.638		
95% CI	−0.99–2.35	−1.25–2		
△	−0.68 ± 3.56	−0.37 ± 3.76	.786	−2.57 to 1.96
Translation (flexion-extension) (mm)				
Preoperative	2.40 ± 1.62	2.03 ± 1.36	.421	−0.55 to 1.29
Final follow up	1.85 ± 1.24	1.81 ± 1.42	.932	−0.79 to 0.86
*P*-value	0.14	0.514		
95% CI	−0.2–1.29	−0.45–0.88		
△	−0.55 ± 1.56	−0.21 ± 1.54	.489	−1.3 to 0.63

a △ indicates difference between the baseline and final follow-up.

## DISCUSSION

**FIGURE 4.**
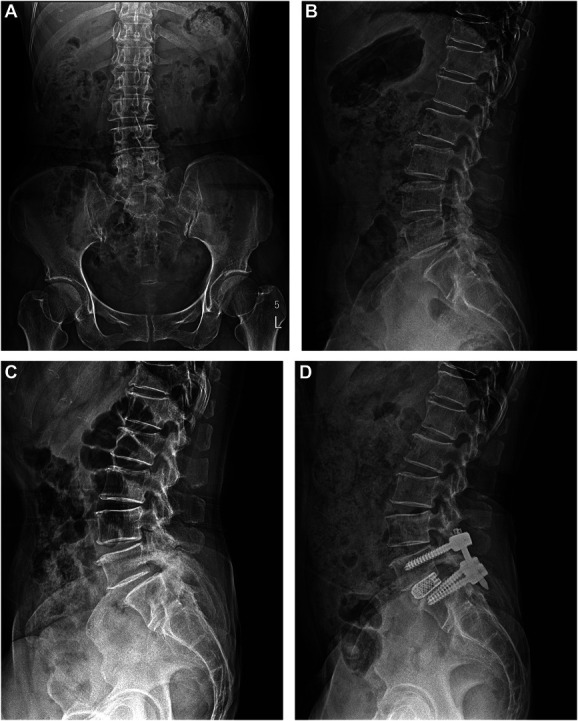
Plain x-ray images of a 61-year-old female who underwent full-endoscopic decompression at the L5/S1 level for spinal stenosis with grade 1 spondylolisthesis and eventually got the fusion surgery after recurrent pain because of progression of the spinal instability. **A**, The preoperative and **B**, postoperative images of endoscopic decompression and **C**, the preoperative and **D**, postoperative images of the fusion surgery show the postoperative progression of degenerative spondylolisthesis.

Based on our study, full-endoscopic decompression may be a good treatment option in patients with LSS and grade I DS even with spinal instability. In our study, the symptoms of patients graded by VAS scores for the legs and back, ODI scores, and Macnab's criteria improved significantly at all follow-up points. The rates of surgical complication and revisions were low (7%). Approximately half of the patients (46.5%) developed an increase in the percentage of slip at the final follow-up. However, progression of DS, defined as an increase in percentage of slip of more than 5 percent, was observed in 7% (three cases) of patients. Two of the affected patients exhibited no symptom aggravation. One patient developed symptoms of spinal instability and eventually underwent fusion surgery (Figure 4). However, it is still uncertain whether the development of instability was due to the surgery. When we evaluated patients in the I group, there was no difference in the clinical outcomes between them and the other group. This finding is consistent with the report of Rampersaud et al,^19^ a retrospective study that indicated that slip progression in DS is not associated with increased functional disability after surgical decompression. Interestingly, the other half of the patients (53.5%) developed a decrease in the percentage of slip, which could be interpreted as an improvement in DS. Furthermore, surgery improved baseline radiological instability in a significant number of patients. The number of patients with preoperative radiological instability was 22, but the number reduced to 12 at the final follow-up after the surgery. This may be cautiously interpreted as “restabilization,” taking into account the fact that there were five patients whose pre-existing instability was aggravated. This restabilization occurred within 1 year. The fact that spinal instability improves after surgery contradicts common sense expectations; decompression surgery has a risk of aggravating instability. One plausible explanation for this observation is that minimally invasive surgery tends to preserve the motion segment and supporting tissues. Scholler et al^20^ reported 12.8% and 3.3% subsequent fusion rates after open laminectomy and microscopic ULBD, respectively, in patients with LSS and DS. Youn et al^14^ reported facet preservation rates of 84% for the ipsilateral side and 91% for the contralateral side in LE-ULBD.

We hypothesized that because full-endoscopic decompression almost completely preserves tensegrity, there is little destructive effect on the stability of the spine, and postoperative changes such as fibrosis may restabilize the unstable part. We cannot necessarily conclude that decreased slippage means “restabilization” because better supporting muscle works from patient's comfort after surgery could make less slippage. But muscle works alone cannot explain the improvement of dynamic instability after surgery.

Our study had some limitations. This was a retrospective study that analyzed only short-term follow-up data. The progression of DS can be symptomatic, and instability may be aggravated in the long term. Furthermore, our study did not compare LE-ULBD with other surgical techniques. This study should be followed by a prospective randomized controlled trial to determine the efficacy and safety of LE-ULBD in patients with LSS associated with grade I DS.

## CONCLUSION

Full-endoscopic decompression may be a good treatment option for patients with LSS associated with grade I DS with or without spinal instability. This surgical technique may restabilize pre-existing instability.

## COMMENTS

In the current retrospective case series, the authors described clinical and radiographic outcomes of lumbar full-endoscopic unilateral laminotomy for bilateral decompression (LE-ULBD) for lumbar spinal stenosis (LSS) with Grade-1 degenerative spondylolisthesis (DS) in 43 patients with 1-year follow-up. Notably, 7% developed slip progression, defined as >5% percentage of body, with one subsequent fusion and two diskectomies. Surprisingly, a sizable subgroup showed decreased postoperative anterolisthesis. This is a well-performed, timely study considering growing endoscopic interests and indications and the high prevalence of LSS from concomitant DS.

The impact of endoscopic decompression on spinal instability warrants further discussion. The authors discussed mixed study results of open surgery, which destroys multifidus muscles and supraspinous/interspinous ligaments compared with lower-risk tubular or endoscopic approaches.^[Bibr R1a][Bibr R2a][Bibr R3a]^ One study showed the multifidus initially atrophies after endoscopic surgery but can be reversed with time and exercise.^4a^ Maximal multifidus preservation likely occurs with unilateral, uniportal endoscopy. Even preoperatively, multifidus atrophy can occur from symptomatic LSS/DS inactivity or posterior ramus dysfunction, exacerbating pain and spondylolisthesis.^[Bibr R5a][Bibr R6a][Bibr R7a]^ Theoretically, LE-ULBD with multifidus preservation and increased postoperative activity could allow improved multifidus strength and dynamic stability at or above preoperative baseline.

Despite endoscopic muscle sparing, decompression is unlikely to improve radiographic stability, necessitating alternative explanations for why 53.5% of patients had decreased (improved) anterolisthesis. Radiographic slip improvement exemplified in Figure 2 could be attributed to slight rotational variation evidenced by differing ASIS alignments between films. Overall, stating LE-ULBD offers adequate neural decompression with the lowest possible risk of iatrogenic instability should be sufficient. Although interesting, we recommend reserving any claims about LE-ULBD improving spondylolisthesis slip.

In the current series, impressively, only 7% developed radiographic anterolisthesis progression with one subsequent fusion. Future studies should compare long-term outcomes and costs between LE-ULBD and instrumentation cohorts. If long-term equipoise exists between fusion revision rates from adjacent segment disease and decompression revision rates, as suggested by Försth and colleagues,^2a^ then endoscopic LE-ULBD may prove a cost-effective, less invasive alternative to fusion for many patients. We commend and thank the authors for their utilization of this progressive technique and the opportunity to review this important, timely study.

**Campbell Liles**

**Raymond J. Gardocki**

**Scott L. Zuckerman**

*Nashville, Tennessee, USA*
